# Artificial intelligence–enhanced microsurgical training: a systematic review

**DOI:** 10.1038/s41746-026-02452-5

**Published:** 2026-02-20

**Authors:** Wameth Alaa Jamel, Mohammed Jameel, Ibrahim Riaz, Yousif F. Yousif, Rocio Perez H, Valeria de la Torre, Ishith Seth

**Affiliations:** 1 Department of Plastic and Reconstructive Surgery, Baghdad Al-Russafa Health Directorate, Baghdad, Iraq; 2 Department of Accident and Emergency, East Lancashire NHS Hospitals Trust, Lancashire, UK; 3 Department of Acute Medicine, Basildon and Thurrock University Hospital, Basildon, UK; 4 Department of Plastic and Reconstructive Surgery, The Royal Marsden Hospital NHS Foundation Trust, London, UK; 5 Department of Plastic and Reconstructive Surgery, Policía Nacional del Perú, Lima, Peru; 6 Department of General Medicine, Distrito Sanitario Poniente de Almería, Almería, Spain; 7 Department of Plastic and Reconstructive Surgery, Peninsula Health, Victoria, NSW Australia

**Keywords:** Computational biology and bioinformatics, Health care, Mathematics and computing, Medical research, Scientific community

## Abstract

Artificial intelligence (AI) offers objective, adaptive tools for skill enhancement in microsurgical training, but evidence is fragmented. This systematic review evaluates AI-enhanced training efficacy compared to traditional methods, focusing on technical performance, learning efficiency, and skill retention. Following PRISMA guidelines, databases (MEDLINE, Embase, Cochrane, IEEE Xplore, Web of Science) were searched from January 2010. Data on study characteristics, AI models, outcomes (time, errors, skill metrics), risk of bias, evidence certainty (GRADE), methodological quality, and reporting quality were extracted and synthesized narratively. From 2,056 records, 13 studies were included, involving 3–50 participants, mostly single-centre with varied designs. AI/ML models, such as Mask R-CNN, YOLOv2, ResNet-50, and other convolutional neural networks, were primarily used for assessment or guidance/coaching, focusing on instrument tracking (30.8%), motion analysis (23.1%), tutoring/guidance (15.4% each). Median accuracy 83.8% (IQR 78.4–88.2%). AI improved technical skills (reduced errors) and learning curves via real-time feedback, with promising retention outcomes. RoB high; evidence certainty very low. Reporting quality high/moderate, external validation poor. AI enhances microsurgical training with objective metrics and personalised feedback, showing promising technical advantages in simulations. However, heterogeneous, low-quality evidence limits generalisability. Future research needs multi-centre RCTs, standardised outcomes, external validation, and ethical considerations for clinical translation.

## Introduction

Microsurgery is an onerous surgical speciality requiring a sharp learning curve. Manual dexterity and pinpoint visual-motor skills define successful outcomes^1–3^. Microsurgery is broadly interpreted in this review as surgical procedures performed under an operating microscope, typically involving manipulation of small structures such as blood vessels (often <3 mm in diameter) or nerves, and spanning multiple specialities including plastic and reconstructive surgery (and supermicrosurgery), ophthalmology, neurosurgery, and others with overlapping technical demands^4^. The nature of these procedures, especially in supermicrosurgery, indicates the need for comprehensive training techniques^3^. Nevertheless, the conventional Halstedian apprenticeship approach of ‘see one, do one, teach one’ is becoming increasingly impractical^5^. Historically, surgical training has been characterised by a lack of formal structure and objective assessment. Systemic pressures such as reduced resident doctor working hours are further testing this model^5,6^. In addition, contemporary training pathways are resource-intensive, often relying on live animal models that have their own ethical and logistical challenges^7^. The intrinsic subjectivity of this apprenticeship model is its greatest weakness; feedback is often informal, unstructured, and highly variable^8,9^. The absence of standardised, reliable evaluation, along with the pressing need for patient safety, means that novel training models need to be used that entail learning new skills on real patients^7,10^. Beyond the limitations of the Halstedian apprenticeship, a specific gap persists in how artificial intelligence is being integrated into microsurgical training. Existing reviews of AI in microsurgery predominantly emphasise clinical applications such as planning, intraoperative assistance, and postoperative monitoring, with only cursory coverage of the training paradigms and learner-centred outcomes. Broader AI‑in‑surgery and AI‑in‑simulation reviews collate microsurgical and non‑microsurgical procedures and do not offer a focused analysis of microsurgical curricula or trainee‑level validation^11–13^. The present review addresses this gap by concentrating specifically on AI systems used with human microsurgical trainees and educational settings, and by systematically appraising the methodological quality (AMSTAR‑2), AI reporting and validation (AITEL), and the extent to which AI‑derived metrics are aligned with established competency frameworks.

Consequently, artificial intelligence (AI) is emerging as a novel, innovative disruption in surgical education. It provides the objective measurements and a formal framework otherwise lacking in conventional models^14,15^. Improvements in machine learning (ML), deep learning, and computer vision make it possible to automatically assess skills using data^16^. AI systems can process an extensive amount of multimodal data rapidly and retain records of crucial metrics. Thus, performance, skills and mistakes can be accurately assessed^17^. Additionally, these systems can offer tailored feedback when integrated with simulation technologies such as Virtual Reality (VR) and Augmented Reality (AR)^14,18^. Thus, there is a possibility to overhaul the current training methodology by providing adaptive and risk-free learning pathways for each learner^14^.

For the purposes of this review, ‘AI-enhanced’ microsurgical training is defined as the integration of artificial intelligence techniques, specifically ML, deep learning, computer vision, or other algorithmic models. These enable objective automated skill assessment, real-time or adaptive feedback, predictive performance modelling, or personalised tutoring/guidance. This distinguishes AI-enhanced approaches from traditional or ‘on-AI’ simulation methods, such as pure VR or AR platforms that offer immersive environments, predefined task scenarios, or static overlays.

Although AI has the potential to transform microsurgical training, there is reason to be cautious. The existing evidence is still in its infancy and lacking coherence. Hence, there is a need for validation of laboratory promise by proving clinical effectiveness^16^. Funke et al. noted significant variability in study techniques, AI models, surgical tasks, and performance indicators in their study^19^. This overall lack of standardisation makes it challenging to make generalisations about how well AI-based technologies actually work. Another key problem is that most suggested AI models lack external validation, and most of them remain in the research stage and have only been evaluated on small, non-generalisable datasets^20^. Therefore, a systematic review is urgently needed to gather, critically evaluate, and integrate these diverse findings.

This review aims to address this gap by evaluating the quality and outcomes of existing studies, identifying potential pathways for clinical translation, and highlighting discrepancies in the evidence. The primary objective of this study is to perform a systematic review of the effectiveness of AI-enhanced microsurgery training in comparison to conventional methods. The secondary objective is to measure enhancements in technical performance by examining factors such as time expenditure, error rates, and evaluation scales. The impact on learning efficiency and skill retention will be examined, along with an analysis of results across pertinent subgroups, including AI modality and trainee experience level. In doing so, this review seeks to move beyond descriptive cataloguing of AI applications and instead provides a critical evaluation of model validation and curricular integration for microsurgical training programmes.

## Results

**Fig. 1:**
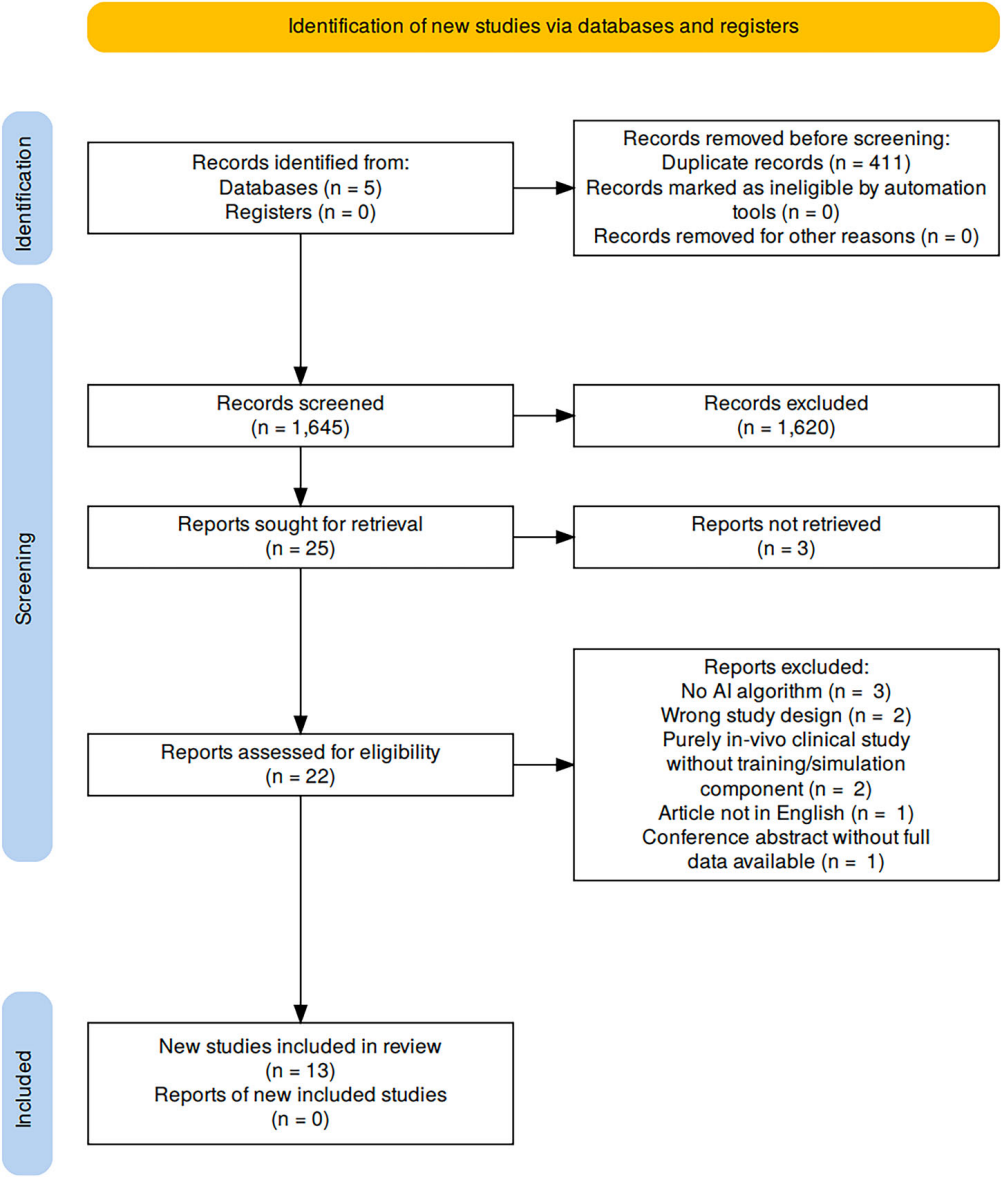
PRISMA flow chart of study selection process. This diagram illustrates the systematic process of identifying, screening, and selecting studies for inclusion in the review.

The database search yielded 2056 records, from which 411 duplicates were removed, and 1645 study abstracts were screened. The selection process narrowed the pool to 25 papers, 3 of which could not be retrieved. Twenty-two records were then subjected to full-text screening. During this stage, 9 studies were excluded because of wrong study design, purely in-vivo clinical study without training/simulation component and the fact that they were published in languages other than English, leaving a total of 13 studies to be included in our review. The study selection process is summarised in Fig. 1 using the PRISMA flow chart.

### Studies characteristics

**Fig. 2:**
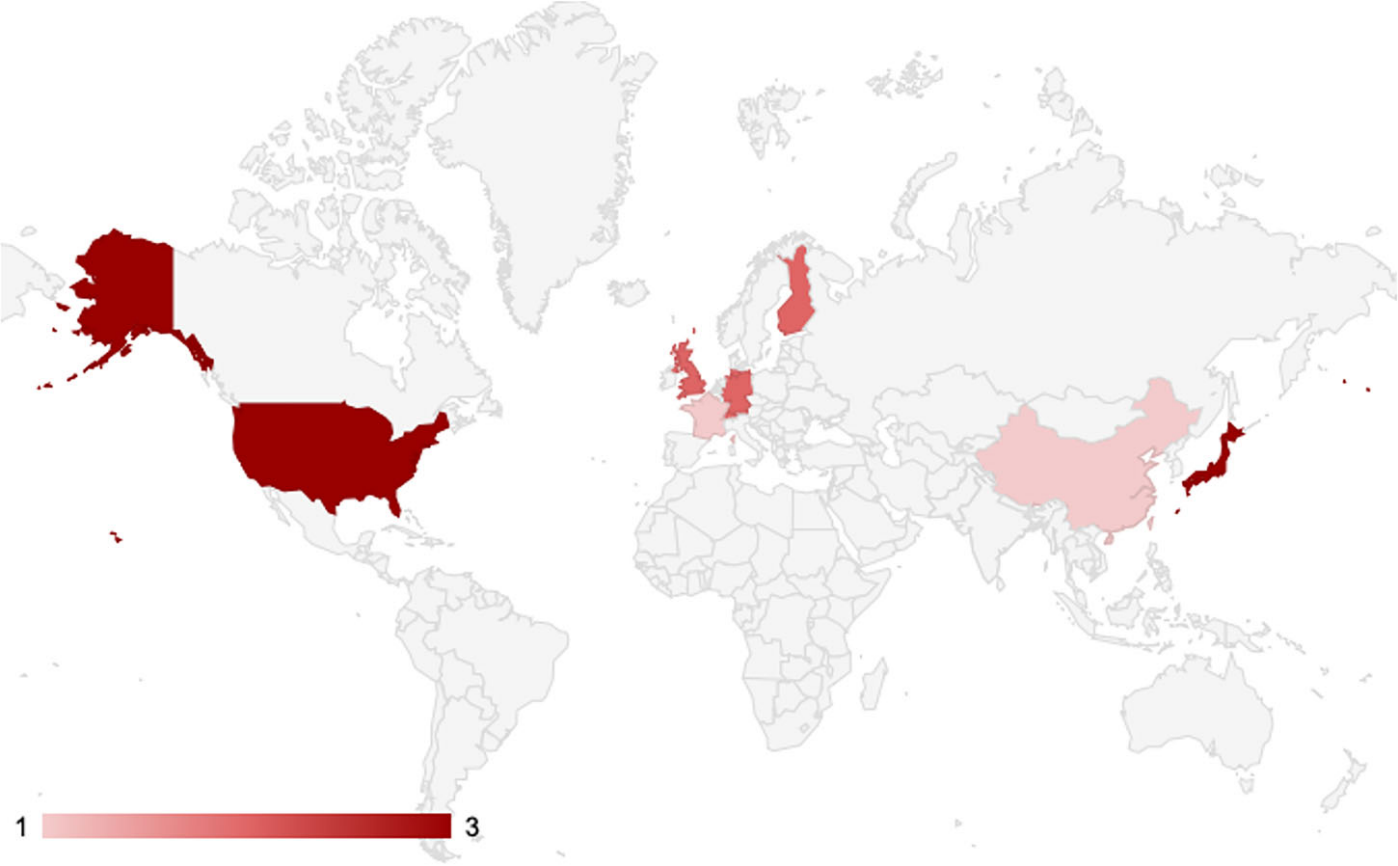
Geographic distribution of included studies highlighted by country. This map illustrates the worldwide spread of the studies incorporated in the review.

The included 13 studies were conducted across eight countries from 2018 to 2025 (Fig. 2). Most were single-centre (*n* = 12), with one multi-centre study. Sample sizes ranged from 3 to 50, with 10 studies involving participants with prior microsurgery experience. Study designs varied, including validation, experimental, development, and simulation studies. AI/ML models (e.g., Mask R-CNN, YOLOv2, ResNet-50, convolutional neural networks) were used mainly for assessment (*n* = 10) or guidance/coaching (*n* = 3), with inputs like video, kinematics, or eye-tracking and outputs such as velocity, path distance, and performance scores. Simulators were used in 10 studies, with two using robotic platforms and one using simulators and wet labs. Validation was primarily conducted through internal cross-validation (10 studies), with two studies utilising external datasets and one employing cross-domain transfer learning. Feedback was post-hoc (8 studies), adaptive (3 studies), real-time (2 studies), or visual (1 study). All studies used expert or standard comparators, with reference standards like Objective Structured Assessment of Technical Skills (OSATS) and Stanford Microsurgery and Resident Training Scale (SmaRT). Explainability was addressed in 10 studies. These findings are summarised in Table 1, and a full description of the studies’ characteristics is found in the Supplementary Table 1.

**Table 1:** Summary of the included studies characteristics

Author (Year)	Country	Sample size	Type of AI/ML model	Input	Outputs	Validation Method	Implementation Type
Davids et al. (2021)^27^	United Kingdom	19	Mask R-CNN	Video	Velocity/Acceleration/Working area/Curvature/Inter-tool distance	Internal CV (AUC and accuracy on internal dataset, Mann-Whitney U test for differences)	Standard Architecture/Open-Source
Khalil et al. (2025)^23^	Finland	11	Modified Long-Range Recurrent Convolutional Networks (LRCNs; three variants)	Video	Phases/Confidence/Time spent	External dataset (generalisation tested across other datasets)	Standard Architecture/Open-Source
Huaulmé et al. (2018)^28^	France and Japan	4	Pattern mining	Video/Kinematics	Sequential signatures/Classification accuracy	Internal CV (validated on internal dataset of 17 tasks, tested for expertise and participant classification)	Custom Research Pipeline
Keller et al. (2020)^24^	USA	3	Deep deterministic policy gradients from demonstrations (DDPGfD)	Video/Kinematics	Needle insertion depth/deformation	Internal CV (trained on ex vivo corneas, evaluated in mock trials against surgical fellows)	Custom Research Pipeline
Koskinen et al. (2022)^29^	Finland	Not reported	Convolutional neural network (YOLOv5l)	Video/Eye-tracking	Path length/Velocity/Acceleration/Jerk/Gaze-tool distances	External dataset (transfer learning; validation on held-out data, testing on partially unseen settings)	Standard Architecture/Open-Source
Gomaa et al. (2024)^25^	Germany	Not reported	Reinforcement (PPO) + imitation (GAIL)	Video/Kinematics	Incision precision/Time/Following technique rate	Internal CV (evaluated in simulation for generic vs. adapted agent trade-offs)	Standard Architecture/Open-Source
Gonzalez-Romo et al. (2023)^30^	USA	6	Convolutional neural network (Mediapipe)	Video	Excess motion/latencies	Internal CV (compared performance across 6 operators with varying expertise)	Standard Architecture/Open-Source
Menozzi et al. (2020)^21^	Switzerland and Taiwan	23	Intelligent Tutoring System (ITS)	Kinematics	Performance score (deviation)	Internal CV (23 trainees, ANOVA for training effects)	Custom Research Pipeline
Ropelato et al. (2020)^22^	Switzerland	50	Intelligent Tutoring System (ITS)	Kinematics	Performance score (deviation)	Internal CV (50 participants, ANOVA for training effects)	Custom Research Pipeline
Stögner et al. (2025)^31^	USA and Germany	Not reported	Deep convolutional neural network	Video	Quantitative assessment (anastomosis metrics)	Internal CV (84 videos, t-test, ANOVA, regression for differences)	Standard Architecture/Open-Source
Zhang et al. (2020)^26^	United Kingdom and China	8	Transfer learning (deep neural networks for skill assessment)	Kinematics	Skill classification/Trajectory/Velocity	Cross-domain transfer learning (from JIGSAWS to MRRPD, LOSO cross-validation)	Standard Architecture/Open-Source
Sugiyama et al. (2024)^32^	Japan	14	YOLOv2	Video	Procedural time/Path distance/Jerk	Internal CV (66 trials from 14 surgeons, correlations with rating scales and experience)	Standard Architecture/Open-Source
Sugiyama et al. (2025)^33^	Japan	14	ResNet-50, YOLOv2	Video	Vessel area fluctuation/Tissue errors/Path distance/Jerk	Internal CV (28 trials from 14 surgeons, correlations with rating scales, discriminant analysis)	Standard Architecture/Open-Source

To further highlight the heterogeneity of AI approaches across the included studies, Table 2 provides a taxonomy categorising the systems by modelling paradigm, primary analysis modality, learning paradigm, and system origin. This classification highlights the research-stage nature of these technologies. Across the included studies, two implementation patterns were evident. First, many systems applied standard deep‑learning backbones or conventional machine‑learning classifiers to microsurgical video or motion data (e.g. convolutional networks and tree‑based models), representing ‘standard architectures’ adapted to the domain. Second, several groups implemented bespoke research pipelines combining custom feature extraction, sensor fusion, and non‑public code, which we categorised as ‘custom research pipelines’.

**Table 2:** Taxonomy of included studies according to modelling paradigm, primary analysis modality, learning paradigm, and system origin

Study	Modelling paradigm	Primary analysis modality	Learning paradigm	System origin
Davids (2021)	Deep learning	Vision models	Supervised models	Self-Developed Research Prototype
Huaulmé (2018)	Classical ML	Kinematic/motion models	Rule-based/adaptive systems	Self-Developed Research Prototype
Khalil (2025)	Deep learning	Vision models	Supervised models	Self-Developed Research Prototype
Gomaa (2024)	Deep learning	Tutoring/decision-support systems	Reinforcement/imitation learning	Self-Developed Research Prototype
Gonzalez-Romo (2023)	Deep learning	Kinematic/motion models	Supervised models	Self-Developed Research Prototype
Menozzi (2020)	Classical ML	Tutoring/decision-support systems	Rule-based/adaptive systems	Self-Developed Research Prototype
Ropelato (2020)	Classical ML	Tutoring/decision-support systems	Rule-based/adaptive systems	Commercial System
Keller (2020)	Deep learning	Vision models	Reinforcement/Imitation Learning	Self-Developed Research Prototype
Stögner (2025)	Deep learning	Vision models	Supervised models	Self-Developed Research Prototype
Koskinen (2022)	Deep learning	Vision models	Supervised models	Self-Developed Research Prototype
Zhang (2020)	Deep learning	Kinematic/motion models	Supervised models	Self-Developed Research Prototype
Sugiyama (2024)	Deep learning	Vision models	Supervised models	Self-Developed Research Prototype
Sugiyama (2025)	Deep learning	Vision models	Supervised Models	Self-Developed Research Prototype

### Risk of Bias, Evidence Certainty, and Reporting

Risk of bias was assessed using RoB-2 for two randomised experimental studies (Menozzi et al., 2020; Ropelato et al., 2020), both yielding high overall risk judgments driven by biases in randomisation and result selection^21,22^. PROBAST was implemented on four prediction model studies (Gomaa et al., 2014; Keller et al., 2020; Khalil et al., 2019, Zhang et al., 2019), all of which demonstrated overall high risk of bias and applicability concerns, with participant selection, outcome determination and analysis found to be particularly problematic^23–26^. QUADAS-2 evaluated the remaining seven validation/diagnostic studies (Davids et al., 2021; Gonzalez-Romo et al., 2023; Huaulmé et al., 2018; Koskinen et al., 2022; Stögner et al., 2025; Sugiyama et al., 2024; Sugiyama et al., 2025), revealing high risk in five (mainly from patient selection, index test, and reference standard domains) and unclear risk in two^27–33^. These evaluations are shown in Tables 3–6.

**Table 3:** RoB-2 assessment of the two included RCTs

Study	Domain 1: randomisation	Domain 2: deviations	Domain 3: missing data	Domain 4: outcome measurement	Domain 5: reporting	Overall
Menozzi 2020	High	Low	Low	Low	Some concern	High risk
Ropelato 2020	High	Low	Some concern	Low	Some concern	High risk

**Table 4:** PROBAST assessment of the prediction model studies

Study	RoB				Applicability			Overall	
	Participants	Predictors	Outcome	Analysis	Participants	Predictors	Outcome	RoB	Applicability
Khalil 2025	—	—	—	—	—	+	—	—	—
Gomaa 2024	—	+	—	—	—	—	—	—	—
Keller 2020	—	+	+	—	—	+	—	—	—
Zhang 2020	—	+	+	—	—	+	+	—	—

*PROBAST* Predication model Risk Of Bias ASsessment Tool, *RoB* Risk of Bias.

(+) indicates low RoB/low concern regarding applicability.

(−) indicates high RoB/high concern regarding applicability.

**Table 5:** QUADAS-2 assessment of the validation/diagnostic studies

Study	Patient selection	Index test	Reference standard	Flow and timing	Risk of bias
Davids 2021	High	High	High	Low	High
Gonzalez-Romo 2023	High	High	High	Low	High
Huaulmé 2018	High	High	High	Low	High
Koskinen 2022	Unclear	Low	Unclear	Low	Unclear
Stögner 2025	Unclear	Unclear	Unclear	Low	Unclear
Sugiyama 2024	High	Unclear	Low	Low	High
Sugiyama 2025	High	Low	Low	Low	High

**Table 6:** GRADE certainty of evidence evaluation of the included studies

Study	Risk of bias^b^	Indirectness^c^	Inconsistency	Imprecision^d^	Publication bias	Overall Certainty
GRADE-DTA assessment						
Sugiyama, 2025	Serious	Serious	N/A	Very serious	Unclear	Very low ^a1,b1,c1,d1^(⊕◯◯◯)
Zhang, 2020	Very serious	Very serious	N/A	Very serious	Unclear	Very low ^a2,b2,c2,d2^ (⊕◯◯◯)
Standard GRADE assessment						
Khalil 2025	Very serious	Very serious	N/A	Very serious	Unclear	Very low^b3,c3,d3^(⊕◯◯◯)
Davids 2021	Very serious	Very serious	N/A	Very serious	Unclear	Very low^b3,c3,d3^(⊕◯◯◯)
González-Romo 2023	Very serious	Very serious	N/A	Very serious	Unclear	Very low^b3,c3,d3^(⊕◯◯◯)
Gomaa 2024	Very serious	Very serious	N/A	Very serious	Unclear	Very low^b3,c3,d3^(⊕◯◯◯)
Menozzi 2020	Serious	Not serious	N/A	Serious	Unclear	Very low^b3,d4^(⊕◯◯◯)
Huaulmé 2018	Serious	Not serious	N/A	Serious	Suspected	Very low^b3,d4^(⊕◯◯◯)
Koskinen 2022	Serious	Not serious	N/A	Serious	Suspected	Very low^b3,d4^(⊕◯◯◯)
Keller 2020	Serious	Very serious	N/A	Serious	Unclear	Very low^b3,c3,d4^(⊕◯◯◯
Ropelato 2020	Serious	Not serious	N/A	Serious	Unclear	Very low^b4,d5^(⊕◯◯◯)
Stögner 2025	Serious	Not serious	N/A	Serious	Unclear	Very low^b4,d3^(⊕◯◯◯
Sugiyama 2024	Serious	Not serious	N/A	Serious	Unclear	Very low^b5,d3^(⊕◯◯◯)

^**a1**^Index combines vessel-area change + kinematic metrics vs blinded scale; promising AUCs but high RoB, indirectness, and imprecision.

^**a2**^Accuracy reported without thresholds; experience-based reference; strong indirectness to clinical skills/performance outcomes.

^**b**^RoB:

^1^small sample; data-driven feature/threshold selection; potential overfitting; unclear blinding.

^2^small sample; non-validated labels; evaluation via LOSO CV; limited DTA reporting.

^3^small sample; model development on same domain; unclear blinding.

^4^potential order/period effects; unclear randomisation of sequence; lack of blinding to condition; performance metrics scored within the same platform.

^5^single-examiner SAMS reference; multi-source convenience videos; partial industry funding; no blinding reported.

^c^Indirectness:

^1^simulation; competence proxy, not clinical outcomes.

^2^robot platform; simulation; categorical ‘skill level’ proxy; domain shift.

^3^outcomes are not patient- or competency endpoints

^d^Imprecision:

^1^*n* = 14; wide CIs.

^2^*n* = 8; no CIs; multiple models/tasks.

^3^ small sample; no effect estimate on improvement.

^4^limited power for between-group difference.

^5^uncertainty about magnitude of benefit despite *n* = 50.

Certainty of evidence was evaluated using the DTA-GRADE approach in two studies (Sugiyama et al.; Zhang et al.), both rated as very low overall due to serious to very serious concerns in risk of bias, indirectness, and imprecision, with unclear publication bias^26,33^. The standard GRADE methodology was applied to the remaining 11 studies (Khalil et al.; Davids et al.; González-Romo et al.; Gomaa et al.; Menozzi et al.; Huaulmé et al.; Koskinen et al.; Keller et al.; Ropelato et al.; Stögner et al.; Sugiyama et al.), all had very low certainty ratings, primarily driven by serious to very serious issues in risk of bias, indirectness (in some), and imprecision, alongside unclear or suspected publication bias^21–25,27–32^.

AMSTAR-2 was used to assess the quality of prior systematic reviews, identifying both critical and non-critical flaws to evaluate overall confidence in their results. Carciumaru et al. and Abdul Saleem et al. were appraised as “critically low” confidence, while Raquepo et al was appraised as “low” confidence^34–36^. Carciumaru et al. identified three critical flaws (items 2, 7, 9) and five non-critical flaws (items 1, 3, 5, 6, 10). Abdul Saleem et al. showed three critical flaws (items 2, 7, 13) and four non-critical flaws (items 3, 6, 10, 14). Raquepo et al. did comparatively better with one critical flaw (item 7) and one non-critical flaw (item 10). Across the three reviews evaluated, the most frequently observed critical items were item 7 (no in all three) and item 4 (partial yes in all three). Our review yielded a higher confidence rating, with no methodological shortcomings, thereby highlighting its reliability for credible scientific conclusions. A summary of the AMSTAR-2 assessment is found in Supplementary Table 2.

**Fig. 3:**
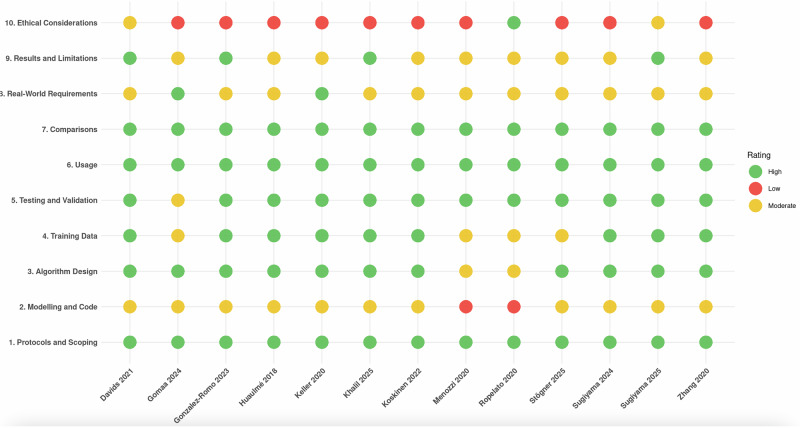
AITEL checklist for reporting quality assessment. The diagram outlines the AITEL checklist criteria applied to assess the reporting quality of the included studies.

Finally, AITEL checklist assessment of the reporting quality across ten domains showed an overall high reporting quality in eight studies and moderate in five. All studies achieved high ratings in protocols and scoping, testing and validation, usage, and comparisons, reflecting strong methodological rigour and practical applicability. Algorithm design was rated high in 11 studies and moderate in two, while training data received high ratings in nine and moderate in four. Modelling and code showed room for improvement, with moderate ratings in 11 studies and low in two, and real-world requirements were moderate in 11 and high in two. Results and limitations were high in four studies and moderate in nine. In contrast, ethical considerations emerged as a notable gap, with low ratings in nine, moderate in three, and high in one. As illustrated in Fig. 3, these patterns highlight strengths in core methodological domains but underscore gaps in ethical reporting and reproducibility, which contribute to the overall low maturity of the field.

In summary, the included studies were predominantly small (3–50 participants), single-centre designs with high risk of bias across all assessed domains (RoB-2, PROBAST, QUADAS-2) and very low evidence certainty. Key gaps included limited external validation and domain-shift testing, poor reproducibility, and near-absence of evaluation for predictive validity, long-term skill retention, or direct clinical outcomes.

### Validation and benchmarking

Across 13 studies, 11/13 reported the reference standard, and none reported inter-rater reliability or a non-inferiority margin. External validation on an independent cohort was reported in 4/13 studies, with 3/13 validating in a different laboratory/institution. Internal validation relied mostly on hold-out splits (8/13), with limited cross-validation (1/13). Domain-shift testing was not frequent (5/13 in total, including specific tests for magnification, platform, or simulation-to-live transfer). Reproducibility practices were low, code was shared in 2/13 studies, datasets were not openly available (1 “upon request”), and no study released model weights. A composite validation-reproducibility readiness score indicated low maturity overall, with a median of 12.5% (IQR 12.5–25.0%) and a maximum of 43.8%. This low overall maturity is consistent with the field’s early research stage, where most studies prioritise internal development over robust external validation and open-science practices.

### Focus areas

**Fig. 4:**
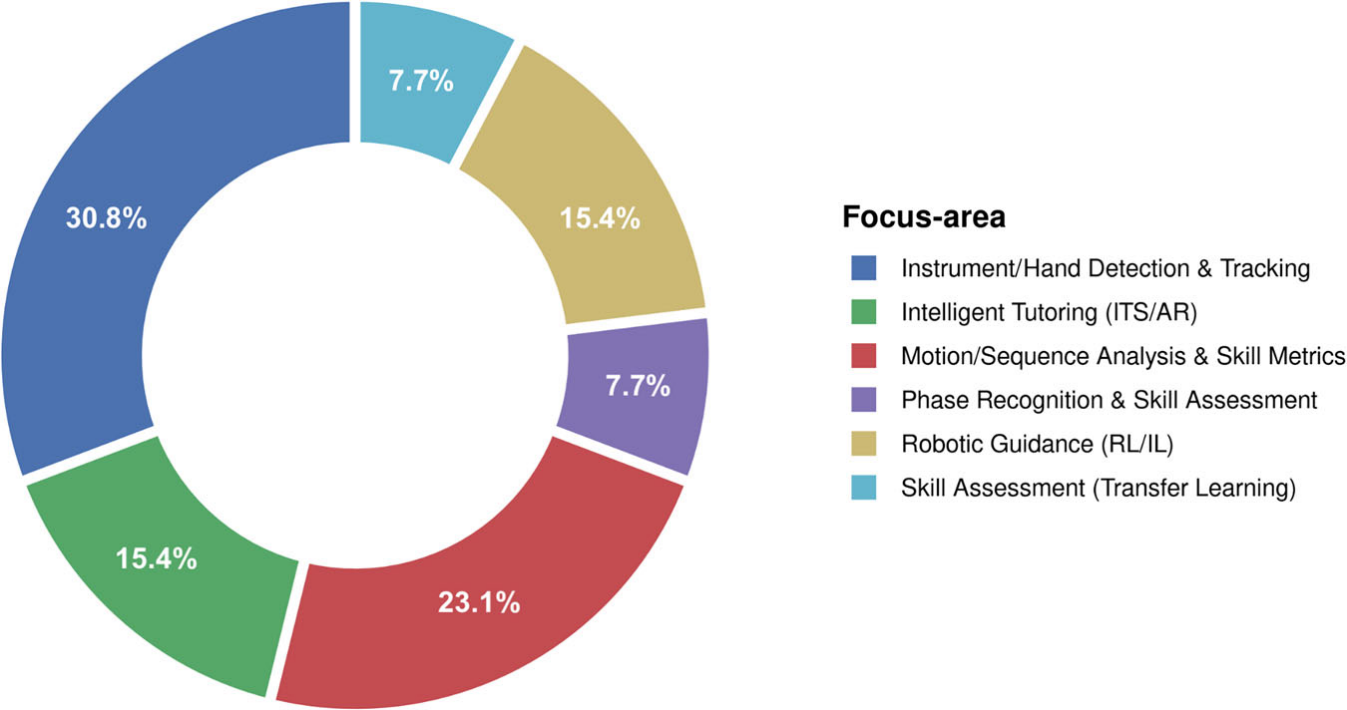
Focus area in the included studies. This chart presents the distribution of primary focus areas across the reviewed studies, categorised by themes.

The distribution of technical focus was led by Instrument/Hand Detection and Tracking (30.8%, 4/13), followed by Motion/Sequence Analysis and Skill Metrics (23.1%, 3/13). Two domains contributed equally at 15.4% each: Intelligent Tutoring (ITS/AR) (2/13) and Robotic Guidance (RL/IL) (2/13). Phase Recognition and Skill Assessment (Transfer Learning) were each represented by a single study (7.7% apiece, 1/13). These categories reflect distinct objectives; for instance, tracking and motion analysis emphasise kinematic outcomes (path distance and jerk indices), while phase recognition focuses on classification accuracy, and RL/IL targets task precision in adaptive guidance; such differences contribute to non-comparable metrics across studies. Collectively, this pattern indicates that most contemporary work emphasises computer-vision–based tracking and kinematic/sequence metrics, with more modest representation of phase-recognition and transfer-learning–based skill assessment; ITS/AR and RL/IL efforts form a substantial secondary tier. These distributions, visualised in Fig. 4, reveal a methodological skew toward detection and analysis tools, which aligns with the patterns of high reporting quality in algorithm design but lower maturity in external validation. Minor deviations from 100% reflect rounding.

### Accuracy

**Fig. 5:**
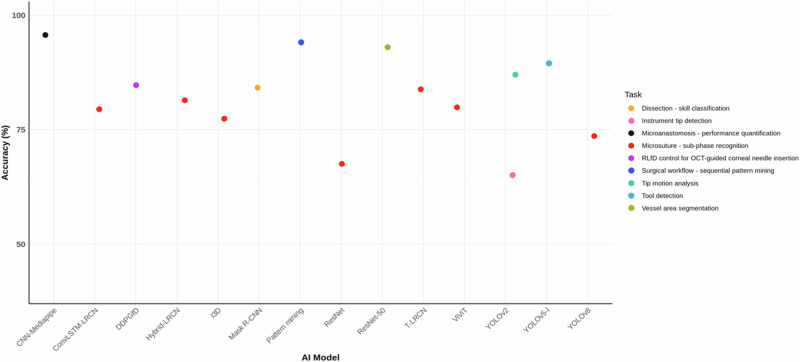
Accuracy across different tasks. This graph displays the comparative accuracy metrics of AI models or interventions across various educational tasks in the included studies.

Across 15 model-task observations, the overall median accuracy was 83.8% (IQR 78.4-88.2%, mean 82.4%). The highest single estimate was 95.7% from CNN-Mediapipe for microanastomosis-performance quantification, while the lowest was 65.0% from YOLOv2 for Instrument tip detection. Microsuture sub-phase recognition (the only task with multiple models; *n* = 7) showed a mean 77.6% and median 79.5% (range 67.6–83.8%), with model-wise accuracies: T-LRCN 83.8%, Hybrid-LRCN 81.4%, ViViT 79.9%, ConvLSTM-LRCN 79.5%, I3D 77.4%, YOLOv8 73.6%, ResNet 67.6%. Single-study point estimates for other tasks were: surgical workflow-sequential pattern mining 94.1% (pattern mining), vessel area segmentation 93.0% (ResNet-50), tool detection 89.5% (YOLOv5-l), tip motion analysis 87.0% (YOLOv2), RLfD for OCT-guided corneal needle insertion 84.8% (DDPGfD), dissection-skill classification 84.2% (Mask R-CNN), and Instrument tip detection 65.0% (YOLOv2). As shown in Fig. 5, accuracy distributions reveal notable performance variability, with higher accuracies (>90%) in tasks like workflow pattern mining and vessel segmentation, likely due to well-defined, static inputs and robust models such as ResNet-50. In contrast, lower accuracies (65–77%) in detection tasks (YOLOv2 for instrument tips) may arise from challenges in real-time tracking, dataset variability, or domain shifts, emphasising the need for improved model robustness and external validation to reduce this variability across applications. Taken together, accuracies were generally high, with microanastomosis quantification, workflow pattern mining, and vessel segmentation showing the strongest single-study performances; however, because most task categories are represented by single studies, cross-task comparisons should be interpreted cautiously. Accuracy across different tasks is shown in Fig. 5.

### Training outcomes and educational impact

Across the 13 studies, outcomes focused mainly on technical skills, showing consistent gains in dexterity, tremor control, and suture precision via automated motion tracking, phase recognition, and kinematic analysis, with reduced path distances, velocities, and jerk indices distinguishing experts from novices^23,26–33^. Cognitive skills like decision-making were less examined but promising in adaptive systems, personalising tasks or using surgeon demonstrations^21,22,25^. Simulator-to-real-surgery transferability was implied through dataset generalisation or ex vivo models but lacked strong clinical validation^24,29,32,33^. Patient safety was indirectly inferred via objective feedback, reducing errors and biases. Educational validity was robust for construct validity and content/face validity in simulations, but predictive validity was underexplored^24,27,28^. AI tools, especially those utilising intelligent tutoring or reinforcement learning, accelerate learning curves compared to fixed methods, with steeper early progress^25,36^. Longitudinal effects were limited, as most studies used single sessions without retention tracking, though some noted sustained gains over short multi-sessions^21,22^. These findings are summarised in Supplementary Table 3.

## Discussion

The aim of this systematic review is to evaluate the effectiveness of AI-enhanced microsurgical training compared to conventional approaches, focusing on improvements in technical performance, learning efficiency, and skill retention. Our findings demonstrate that AI integration, particularly through machine learning models for instrument tracking, motion analysis, and intelligent tutoring systems, enhances technical skill assessment by providing objective metrics such as reduced path distances, velocities, and error rates, while accelerating learning curves and offering adaptive, real-time feedback that surpasses the subjective and unstructured nature of conventional apprenticeship models^14,21,22,25,27–33^. However, the evidence remains heterogeneous in terms of study designs, AI modalities, or outcome measures, including conceptual variability from combining prediction model development (e.g., for automated skill metrics) with training effectiveness studies. This reflects the field’s nascent stage, where algorithmic innovation often supports educational goals. This heterogeneity limits the ability to synthesise overall conclusions, as differing objectives and metrics are often incomparable. Motion-based outcomes like reduced jerk indices or path distances (common in tracking/motion analysis studies) cannot be directly equated to phase-classification accuracies or reinforcement-learning task precisions. Such variations arise from modality-specific goals (e.g., kinematic optimisation in motion analysis vs. sequential pattern recognition in phase studies), precluding quantitative pooling. Future work should consider subgroup analyses to mitigate such heterogeneity. Additionally, the improvements in path length, jerk, suture time, or motion economy represent surrogate technical metrics in simulated environments and should not be interpreted as validated predictors of real surgical performance. Long-term skill retention, transferability to live surgery, and clinical outcomes remain largely unevaluated, with many studies limited to single-session assessments or minimal follow-up.

A key characteristic of AI-enhanced microsurgical training is the wide diversity of approaches, ranging from simple computer vision-based instrument tracking and kinematic analysis (using standard video and modest computing) to advanced intelligent tutoring systems, reinforcement/imitation learning for robotic guidance, and augmented reality integrations (requiring specialised hardware, multimodal datasets, and robust infrastructure). This technological heterogeneity could explain the variability in outcomes and readiness for clinical adoption. Accordingly, there is no single “AI solution,” but a spectrum of tools at different maturity levels, each with distinct data needs, implementation barriers, and scalability challenges.

The strengths of AI applications in microsurgical training are evident in their ability to deliver consistent improvements in key technical domains, as illustrated in Fig. 4. Instrument/hand detection and tracking and motion/sequence analysis and skill metrics (combined ~54% of studies) predominate, using computer vision models to quantify kinematic parameters such as path length, velocity, tremor, and jerk, reliably distinguishing expert from novice performance and improving dexterity and precision^23,26^. It's integrated with AR/VR (~15%) provide adaptive real-time feedback and personalised task sequencing, accelerating learning curves beyond traditional mentorship, which could address the long-standing limitations of traditional models, such as subjective evaluations and unstructured guidance^11,37,38^. Robotic guidance via reinforcement and imitation learning (~15%) enables semi-autonomous assistance in precise tasks, transferring expert demonstrations effectively. Phase recognition and transfer learning-based skill assessment (~15%) offer granular evaluation of surgical sub-phases with potential for longitudinal tracking. Collectively, these modalities deliver objective, reproducible metrics and personalised feedback that could outperform conventional training in technical proficiency.

Despite these strengths, the current evidence on AI-enhanced microsurgical training is restricted by various constraints, including small sample sizes, predominantly single-centre designs, and methodological weaknesses that contribute to a high risk of bias and very low certainty of evidence. This very low certainty implies that future higher-quality research may substantially alter the current estimates of AI’s effectiveness in microsurgical training. Furthermore, all 13 studies exhibited high overall RoB per RoB-2, PROBAST, and QUADAS-2. This, combined with very low GRADE certainty, significantly constrains the strength of our conclusions. For instance, while narrative synthesis suggests AI enhances technical proficiency through reduced path distances and errors and efficiency through objective feedback, these findings must be interpreted cautiously; they are based on methodologically weak studies that may overestimate benefits and lack generalisability, potentially leading to unreliable translation into a clinical training programme.

A significant lack of external validation is observed, as the majority of studies rely on internal cross-validation and hold-out splits instead of utilising independent cohorts or cross-institutional assessments. This, combined with inadequate reproducibility practices, such as infrequent sharing of code, datasets, or model weights, results in low overall maturity scores. Additionally, the absence of standardised outcomes hinders generalisability.

While many studies establish construct validity through distinguishing novice from expert performance via kinematic metrics, predictive validity, linking these surrogates to improved real-world surgical proficiency or patient outcomes, is rarely demonstrated, reflecting the need for more rigorous, longitudinal evaluations^39–41^. To address these, future studies should incorporate a priori sample size planning, multi-centre collaborations for greater generalisability, pre-registered analysis plans on platforms like PROSPERO or Open Science Framework to reduce bias, and routine external validation on independent datasets with domain-shift testing.

For programme directors and training laboratories, the current landscape can be considered in three broad categories of adoptability. First, systems embedded within commercial simulators or proprietary training platforms can, in principle, be implemented through the purchase and integration of those platforms with minimal local AI engineering; this includes tools that provide automated motion metrics and composite performance scores within established devices. Typical requirements include purchase or lease of an AI-enabled microsurgical simulator or AR/VR training platform, a standard high-performance workstation, and institutional IT support. Indicative capital costs are in the high five-figure to low six-figure USD range, with ongoing annual software licensing and maintenance fees. No in-house model development is required, and faculty training focuses mainly on interpretation of automated metrics rather than algorithmic oversight. Second, systems built on standard open‑source architectures (e.g., convolutional models applied to training videos, machine‑learning classifiers on simulator kinematics) are realistically achievable for centres that can access basic institutional data‑science support or external academic partners. Minimum infrastructure includes a modern workstation with a mid-range GPU (e.g., ≥8–12 GB VRAM), secure local storage for video/kinematic data, and access to open-source ML frameworks. Indicative setup costs are in the low five-figure USD range, primarily driven by hardware acquisition. These systems typically require part-time data-science or engineering support, either in-house or through academic collaboration. Third, highly bespoke pipelines that integrate multi‑modal sensing or complex feature engineering typically require dedicated AI expertise and sustained research collaboration, and are likely to remain concentrated in specialised centres until more turnkey solutions become available. Infrastructure commonly includes dedicated GPU servers or cloud compute credits, synchronised multi-camera capture, optional eye-tracking hardware, large-capacity secure storage, and sustained specialist personnel (AI engineers and research staff). Indicative costs are in the mid-five-figure to six-figure USD range or higher, depending on scale and duration, reflecting their primary role as research and development platforms rather than turnkey educational tools. Drawing on the infrastructure described across the included studies, a pragmatic minimum configuration for AI‑assisted microsurgical training consists of: (i) a simulator or bench‑based training setup capable of exporting video and/or motion data; (ii) a secure storage solution for these recordings with appropriate governance; and (iii) a modern workstation capable of handling offline analyses and, where deep‑learning methods are used, a mid‑range GPU to accelerate model training and inference. Beyond this baseline, centres wishing to pursue more advanced applications can consider a ‘research‑ready’ configuration that includes dedicated GPU servers, synchronised multi‑camera video capture, and optional eye‑tracking hardware, which have been used to explore attention, cognitive load, and gaze patterns in microsurgical tasks. Importantly, these additional components should be viewed as enablers for specific research and advanced tutoring applications rather than as mandatory requirements for initial curricular integration. Across both the included studies and related work in surgical education, three integration models are emerging. First, AI is often deployed as an offline assessment layer, applied to recorded training videos or simulator logs to generate objective metrics and structured feedback that complement faculty ratings. Second, some commercial and research platforms embed AI as an intelligent tutoring system, providing real‑time prompts, adaptive task progression, or automated coaching during day‑to‑day training sessions. Third, advanced multi‑modal systems that combine motion, gaze, and contextual information are primarily used as research and benchmarking tools to characterise expertise and cognitive workload, with limited current penetration into routine curricula. For most microsurgical programmes, starting with offline assessment represents the most feasible near‑term integration path. To support pragmatic implementation, a phased adoption pathway can be envisaged. In an initial phase, programmes can prioritise objective motion and time‑based metrics already provided by simulators or simple offline analyses (e.g., path length, economy of motion, idle time), explicitly mapping these metrics to domains within established scales such as OSATS and SMaRT (e.g., economy of motion and path length to ‘time and motion’, jerkiness and tremor to ‘instrument handling’, idle periods to ‘flow of operation’). A second phase involves incorporating AI‑enhanced assessment tools that classify performance level (e.g. novice versus expert) or flag specific error patterns from video and kinematic data, again anchoring outputs to familiar rating domains and using them primarily for formative feedback. In a third phase, centres with access to more advanced platforms or collaborations can integrate intelligent tutoring systems that deliver real‑time guidance and adaptive task difficulty, aligning AI‑generated recommendations with competency milestones and entrustable professional activities rather than creating a parallel assessment framework.

The adoption of AI in microsurgical training faces multiple barriers, which include ethical concerns regarding data privacy, algorithmic bias, accountability, and risks of surgeon autonomy loss through over-reliance on AI systems, and restricted access to diverse high-quality training data^42–44^. Specific to microsurgical training, many AI applications depend on video recordings of trainee performances for instrument tracking and skill quantification. This introduces critical questions around data ownership, trainee consent for video use in model development, and potential privacy risks if videos are stored, shared, or re-identified. Furthermore, the predominance of small, single-centre datasets in the included studies, often drawn from homogeneous trainee cohorts, heightens the risk of algorithmic bias. Such models may unfairly penalise or advantage certain trainees based on factors unrelated to true skill (e.g., institutional techniques, baseline dexterity variations, or underrepresented demographics), exacerbating inequities in assessment and feedback. The implementation of AI systems faces additional challenges due to their high costs associated with specialised hardware and software development, integration with current curricula, and ongoing maintenance needs, particularly in resource-limited settings such as low- and middle-income countries or smaller training programmes^45–47^. Opportunities for advancement require a focus on conducting multicenter RCTs for better evidence quality, to adhere to standardised benchmarking tools like AITEL checklists for reporting and evaluation, to improve AI explainability for better transparency and trust and to enhance reproducibility through open sharing of code, datasets and model weights^48,49^. Finally, the integration of AI with robotic systems and VR/AR platforms enables the development of interactive training environments that could address the preclinical chasm through evidence-based validation and an equal access programme^50–52^.

Furthermore, the GRADE assessments indicated unclear or suspected publication bias in all included studies, compounded by small sample sizes and overwhelmingly positive findings (e.g., AI improvements reported in 92.3% of studies). This raises the possibility of selective reporting, where studies with negative or null results may remain unpublished, potentially overestimating AI’s efficacy. To mitigate this in future reviews, we recommend broader searches for grey literature, trial registries, and unpublished datasets, though our protocol’s focus on peer-reviewed sources aligns with standard systematic review practices.

A critical limitation highlighted by this review is the poor reproducibility of the included studies, with only two studies sharing code and none making datasets publicly available. This severely hampers independent verification, iterative model improvements, and broader adoption in microsurgical training. To address this, future research must prioritise open science practices, including mandatory sharing of code, anonymized datasets, and pre-trained model weights via platforms like GitHub or Hugging Face. Such measures would facilitate external validation across diverse cohorts, reduce duplication of efforts, and promote equity by enabling resource-limited institutions to build upon existing work, ultimately accelerating the translation of AI from experimental prototypes to standardised educational tools.

It is important to note that the evidence synthesised in this review reflects an era dominated by task‑specific models built on convolutional architectures, engineered kinematic features, and classical machine‑learning algorithms. None of the included studies employed large multimodal foundation models or general‑purpose video‑language systems, even though such models are now beginning to appear in broader surgical video understanding and training research.

Looking ahead, multimodal foundation models (large language models coupled with vision and video understanding components) may alter the implementation landscape for microsurgical training. By accessing such models via cloud‑based application programming interfaces, centres without dedicated AI teams could, in principle, leverage pre‑trained capabilities for phase recognition, error detection, and performance summarisation while delegating the underlying engineering to external providers. These systems could also support more natural educational interactions, offering conversational tutoring, case‑based feedback, and structured error debriefing that reference specific segments of trainees’ videos, thereby integrating video understanding, motion analysis, and textual explanation within a single unified framework. The discussion of multimodal foundation models and large language models remains speculative, as none of the 13 included studies employed these technologies. Although such models are increasingly used in broader surgical video analysis and general medical education, no evidence from the reviewed literature directly supports their application in microsurgical training to date. The systems reviewed here generally required intensive local engineering, including the collection and annotation of microsurgical datasets, bespoke feature extraction, and ongoing model training and maintenance. In contrast, general‑purpose multimodal models offer the prospect of more ‘plug‑and‑play’ deployment, allowing educators to concentrate on curricular design and governance rather than algorithm development. However, even if the engineering threshold decreases, the need for robust validation—encompassing construct, concurrent and predictive validity, generalisability across institutions, and safeguards against bias—will remain paramount before such systems can be used for high‑stakes assessment or clinical translation.

In conclusion, AI integration into microsurgical training holds significant but preliminary implications for surgical education by potentially providing access to high-quality, objective instruction with precise and unbiased training content that helps them develop better surgical skills despite limited practice time and ethical concerns regarding animal models. However, the field remains highly experimental, with limited external validation, generalisability, and reproducibility in the current evidence base, as evidenced by the low median validation-reproducibility readiness score of 12.5%, small samples, high overall risk of bias, and insufficient long-term outcomes. The achievement of this potential will require immediate solutions to current challenges, including excessive AI dependence that could threaten surgeon decision-making skills and exacerbate resource inequality in underserved areas. Future research should prioritise multicenter RCTs with long-term follow-ups and adequate power (via sample size planning), pre-registered protocols, along with creating standardised benchmarking and explainability protocols, and exploring multimodal AI platforms that may incorporate federated learning and extended reality and instructor-free systems to eventually achieve clinical practice scalability and equity.

## Methods

This systematic review is conducted in accordance with the Preferred Reporting Items for Systematic Reviews and Meta-Analysis (PRISMA) guidelines^53^. The protocol was registered a priori with International Prospective Register of Systematic Reviews (PROSPERO) under the identifier CRD420251122571.

### Search strategy

A comprehensive electronic search was conducted across MEDLINE (PubMed), Embase, Cochrane Library, IEEE Xplore, and Web of Science from January 1, 2010, onward to identify relevant studies.

The searches were built to optimise accuracy and precision of results. This was done through testing terms and search syntax. Search terms used included: “artificial intelligence”, “machine learning” “deep Learning” “neural networks” “augmented reality” “virtual reality”, “microsurgery”, “simulation training”. Boolean operators were used to combine terms across domains.

### Eligibility criteria

Included studies were randomised controlled trials (RCTs), controlled before-and-after studies, cohort studies with a comparator group, crossover trials or mixed methods with quantitative outcomes. Participants were surgical trainees (residents, fellows, registrars) and novice surgeons in microsurgery trained in any speciality. The interventions consisted of the use of AI-based tools with machine learning and algorithmic elements in hands-on microsurgery training. The control group were exposed and not simulated using AI, that is traditional training. The most important endpoint (skill performance or learning efficiency) should be reported in studies, and secondary endpoints could also be mentioned. All the included studies were required to be published in the English language and presented in a peer-reviewed journal.

Studies were excluded if they were case reports or series without comparators, expert opinions, narrative reviews, protocols or editorials and animal- or cadaver-only studies that did not report human trainee data, did not provide adequate information about AI algorithms or outcomes. This exclusion criterion was applied to ensure direct relevance to human surgical training outcomes, focusing on skill acquisition in trainees; however, studies using simulators, ex vivo models/wet labs, or robotic platforms were included if they involved human participants (surgical trainees or novice surgeons) and evaluated AI’s impact on their microsurgical performance, as these setups provide indirect but valuable insights into clinical applicability. Abstracts or posters without full data were excluded as well.

### Study selection

The search results were uploaded into Rayyan (Qatar Computing Research Institute, Doha, Qatar) (version 3.0) for screening. Two independent reviewers (W.A.J. and R.P.) manually screened the search results to identify studies that met the inclusion criteria. Titles and abstracts were initially reviewed, and if insufficient information was available to determine eligibility, the full article was retrieved for further assessment. Discrepancies in study selection were resolved through consensus between the two reviewers. If disagreements persisted, a senior author (I.S.) was consulted to make the final decision

### Data extraction/synthesis

Two independent reviewers (W.A.J. and M.J.) used a predesigned template on Microsoft Excel (Microsoft Corp., Redmond, WA, USA) (version 16.54) to collect information from the included studies, with discrepancies resolved through consensus; if unresolved, a senior author (I.S.) was consulted. The data extracted included first author, year of publication, number of participants (and breakdown by experience level), specialty, training background, type of AI/ML model, feedback style (real-time, post-hoc, adaptive tutoring, predictive modelling, etc.), external validation (cross-validation, independent dataset), explainability/interpretability of AI output, simulation type, assessment of technical skill (time to task completion, error counts, path length/trajectory length tremor/deviation intensity and motion smoothness) and, if reported, skill retention on follow-up.

### Assessment of risk of bias, evidence certainty, and reporting

The risk of bias was evaluated using tools tailored to the respective study designs. RCTs were assessed with the Cochrane Risk of Bias 2.0 (RoB 2.0) tool^54^. The Prediction model Risk Of Bias Assessment Tool (PROBAST) tool was applied to evaluate prediction model studies, while Quality Assessment of Diagnostic Accuracy Studies, version 2 (QUADAS-2) was used for diagnostic accuracy studies^55,56^. Assessments were performed independently by two reviewers (I.R. and Y.Y.), and any disagreements were resolved by consensus or through consultation with a senior reviewer (I.S.).

A similar strategy was applied when assessing the certainty of evidence, with both GRADE-DTA and the standard GRADE approach utilised^57–59^.

The methodological quality of our systematic review was critically appraised using the AMSTAR-2 tool (A MeaSurement Tool to Assess Systematic Reviews-2)^60^.

Finally, to assess reporting quality across all included studies, the Artificial Intelligence Technology Enhanced Learning (AITEL) checklist was applied^61^.

## Supplementary information

Supplementary Tables

## Data Availability

The datasets generated and/or analyzed during the current study (including template data collection forms and data extracted from included studies) are not publicly available due to not being deposited in a public repository, but are available from the corresponding author on reasonable request.
