# Supplementary material for: Artificial intelligence–enhanced microsurgical training: a systematic review
Source: NPJ Digit Med. 2026 Feb 20;9:267. doi: 10.1038/s41746-026-02452-5 (PMC13036052; doi:10.1038/s41746-026-02452-5)
Supplement: Supplementary file 1 — Supplementary Tables [file 41746_2026_2452_MOESM1_ESM.pdf]

[Supplementary Table 1: Detailed Study Characteristics.]

Author (Year)	Country	Design	Single/Multicenter	Sample size	Prior microsurgery	Duration of study/training sessions	Specialty
Davids et al. (2021)	United Kingdom	Validation study	Single-center	19	Yes	Single session per participant (one recorded arachnoid dissection procedure after brief acquaintance with tools/model; no repetitions or multi-session training)	Neurosurgery
Khalil et al. (2025)	Finland	Experimental development and testing study	Single-center	11	Yes	Data collection only (12 sutures per participant under two magnifications; no multi-session training or repetitions for skill improvement)	Neurosurgery
Huaulmé et al. (2018)	France and Japan	Validation study	Single-center	4	Yes	Not reported	Neurosurgery
Keller et al. (2020)	USA	Validation study	Single-center	3	Yes	20 successful demonstrations (from fellows); 150 insertion episodes (for reinforcement learning)	Ophthalmology
Koskinen et al. (2022)	Finland	Development and case study	Single-center	Not reported	Yes	Not reported	Neurosurgery
Gomaa et al. (2024)	Germany	Simulation study	Single-center	Not reported	Yes	Curriculum learning (low to high polygon complexity); 5M episodes (PPO/RL), 500K episodes (GAIL/IL)	Ophthalmology
Gonzalez-Romo et al. (2023)	USA	Development and comparative study	Single-center	6	Yes	Single session per operator (600 seconds for 4 nonexperts, 180 seconds for experts; multiple bites per session)	Neurosurgery
Menozzi et al. (2020)	Switzerland and Taiwan	Experimental study	Single-center	23	No	5 days (10 minutes per day; participants underwent training sessions with performance recorded before and after each session)	Ophthalmology
Ropelato et al. (2020)	Switzerland	Experimental study	Single-center	50	No	Two training sessions (one with ITS adapting task sequence to progress, one	Ophthalmology

						with fixed sequence; no further duration details)	
Stögner et al. (2025)	USA and Germany	Development and assessment study	Multicenter	Not reported	Yes	Not reported	Plastic and Reconstructive Surgery
Zhang et al. (2020)	United Kingdom and China	Development and validation study	Single-center	8	Yes	Not reported	Not specified
Sugiyama et al. (2024)	Japan	Development and validation study	Single-center	14	Yes	Not reported	Neurosurgery
Sugiyama et al. (2025)	Japan	Validation study	Single-center	14	Yes	Not reported	Neurosurgery

[Supplementary Table 2: AMSTAR-2 quality assessment.]

AMSTAR-2 Criterion	Our Review	Carciumaru et al. (2025)	Abdul Saleem et al. (2025)	Raquepo et al. (2025)
1. PICO component	Yes	No	Partial Yes	Partial Yes
2. Pre-established protocol	Yes	No	No	Yes
3. Explanation of included studies' design	Yes	No	No	Partial Yes
4. Comprehensive search strategy	Yes	Partial Yes	Partial Yes	Partial Yes
5. Duplicate study selection	Yes	No	Partial Yes	Yes
6. Duplicate data extraction	Yes	No	No	Yes
7. List of excluded studies and justification	Yes	No	No	No
8. Description included studies	Yes	Yes	Partial Yes	Yes
9. Assessment of RoB in included studies	Yes	No	Partial Yes	Yes
10. Funding sources	Yes	No	No	No
11. Use of appropriate statistical methods	N/A	N/A	N/A	N/A
12. RoB impact on synthesized results	N/A	N/A	N/A	N/A
13. Results interpretation with RoB reference	Yes	Yes	No	Partial Yes
14. Heterogeneity explanation	Yes	Yes	No	Yes
15. Publication/small study bias investigation	N/A	N/A	N/A	N/A
16. Conflict of interest declaration	Yes	Yes	Yes	Partial Yes
**Critical Flaws**	0	3 (items 2, 7, 9)	3 (items 2, 7, 13)	1 (item 7)
Noncritical Flaws	0	5 (items 1, 3, 5, 6, 10)	4 (items 3, 6, 10, 14)	1 (item 10)
**Overall Rating**	**HIGH Confidence**	**CRITICALLY LOW Confidence**	**CRITICALLY LOW Confidence**	**LOW Confidence**

[Supplementary Table 3: Training Outcomes & Educational Impact.]

Study	Taxonomy of Outcomes					Learning Curve Analysis	Longitudinal Effects
	Technical Skills	***Cognitive Skills***	***Transferability***	***Patient Safety***	***Educational Validity***		
**Davids 2021**	Improved dexterity and precision via lower dissector velocity and inter-tool tip distance.	Not addressed.	Potential from simulator to real microneurosurgery, no direct validation.	Indirect via objective feedback reducing errors.	High construct (AUC 0.977); content/face in high-fidelity model; predictive not assessed.	AI feedback could accelerate vs. traditional observation.	One-time sessions; no retention tracking.
**Khalil 2025**	Better efficiency in suturing phases; distinguishes skill levels via confidence and time metrics.	Indirect via sub-phase recognition.	Good generalization to external datasets; simulator to real potential.	Reduces subjectivity for personalized feedback.	Construct differentiates novice/expert; content in fine phases; face in simulation board; predictive implied.	AI enhances multi-metric assessment faster than traditional.	Primarily one-time; some intra-subject analysis.
**Gomaa 2024**	Precision in cataract incision; adapts to surgeon preferences.	Adapts to unique techniques and planning.	From simulation to physical robots via transfer learning.	Enhances precision, reduces errors with surgeon-in-loop.	Construct evaluates adaptation trade-offs; content in incision phase; face in 3D simulation; predictive not assessed.	RL/IL with curriculum accelerates vs. pure RL.	One-time agent training; no human retention.
**Gonzalez-Romo 2023**	Economy/amplitude/flow of motion; lower excess in experts.	Not addressed.	Simulator to real inferred, no test.	Quantitative reduces bias for safer training.	Construct differentiates levels; content in video phases; face in lab setup; predictive not assessed.	Quantitative feedback accelerates vs. subjective.	One-time simulations; no retention.
**Menozzi 2020**	Motor skills in ILM peeling; significant improvements.	ITS aids adaptive planning.	From AR to physical non-AR setup.	Improves training efficiency for safer procedures.	Construct via ANOVA; content in ILM steps; face in AR; predictive not assessed.	ITS steeper progress vs. fixed sequence.	Over 5 days; no long-term beyond study.
**Huaulmé 2018**	Sequential signatures differentiate expertise.	Captures behavioral patterns.	Simulated to real workflow potential.	Enhances CAS for reduced variability.	Construct high accuracy; content in activities; face in participant data; predictive high for expertise.	Signatures inform personalized training.	One-time tasks; no retention.
**Koskinen 2022**	Kinematics differentiate actions; gaze-tool distances.	Linked to attentional strategies.	From simulated to real OR videos.	Monitors for inefficient movements.	Construct via tests; content in 17 tools; face in case study; predictive implied.	Pipeline supports faster feedback.	Two sessions; no long-term retention.
**Keller 2020**	Needle insertion accuracy outperforms fellows.	Learns from demonstrations for planning.	Ex vivo to real via OCT guidance.	Minimizes deformation/errors in DALK.	Construct superior precision; content in incision; face in ex vivo; predictive generalized policy.	RL from demo accelerates vs. cloning.	Agent learning; no human retention.
**Ropelato 2020**	Micromanipulation scores improve, especially with ITS.	ITS adapts sequences for	AR for ophthalmic; potential to real.	Reduces constraints for safer training.	Construct differentiates modes; content in ILM; face in AR; predictive not assessed.	ITS steeper in second session vs. fixed.	Two sessions; no long-term.

		decision-making.					
**Stögner 2025**	Trajectory length correlates with efficiency.	Not addressed.	Robotic/conventional; real-time potential.	Objective for safer skill assessment.	Construct correlates with SAMS; content in motion; face in videos; predictive not assessed.	Monitors progress over traditional.	Multiple videos; no retention.
**Zhang 2020**	High accuracy in skill classification from motion.	Not addressed.	Cross-domain JIGSAWS to RAMS; real tracking.	Objective metrics/feedback reduce biases.	Construct high accuracies; content in tasks; face in real-time; predictive not assessed.	Transfer learning accelerates with limited data.	Database trials; no retention.
**Sugiyama 2024**	Time/path/jerk correlate with experience; smoother in experts.	Not addressed.	Artificial to clinical potential.	Enhances training to reduce errors.	Construct high ROC; content in phases; face high accuracy; predictive for skill.	Estimates curve in future repeated sessions.	One-time; recommends for efficacy over time.
**Sugiyama 2025**	Vessel fluctuation/deformation/path/jerk correlate with skills.	Not addressed.	Clinical potential with dual AI.	Captures tissue handling for safety.	Construct strong correlations/ROC; content in deformation/motion; face high reliability; predictive combines parameters.	AI feedback enhances curve; future repeated.	No retention; evaluate repeated for curve.

[Supplementary Table 4: Clinician-Focused Summary of Key Findings on AI in Microsurgical Training.]

Key Finding	Explanation	Key Examples	Benefits for Training	Common Outcomes	Limitations
AI Models Used	Computer programs that analyze videos or movements to assess or guide surgical skills, like detecting tools or predicting errors.	- Video analysis (e.g., tracking instrument paths in 8 studies).<br>- Motion metrics (e.g., speed, smoothness in 5 studies).<br>- Guidance systems (e.g., real-time feedback in 3 studies).	Provides objective feedback without needing constant expert supervision.	- Reduced errors (69% of studies)<br>- Faster skill improvement (54%)<br>- Better retention (limited data, 23%).	Models often untested in real surgeries; High setup costs.
Outcomes Measured	What was evaluated: Time to complete tasks, mistakes made, skill levels.	- Technical performance (all 13 studies).<br>- Learning efficiency (8).<br>- Skill retention (3).	Personalized coaching adapts to trainee needs.	AI groups often outperformed traditional methods in simulations.	Results may not apply to all specialties or real operations
Evidence Quality	Overall reliability: mostly low due to small studies and biases.	- High bias risk (all studies).<br>- Very low certainty (GRADE).	Highlights need for better research.	Promising but preliminary advantages in simulations.	Needs larger, multi-center trials for real-world proof.
Recommendations for Clinicians	Begin with simple tools; Scale up with resources.	Phased adoption: start with basic metrics, add AI assessment, then full tutoring.	Improves training equity, especially in low-resource areas.	Objective, repeatable feedback.	Ethical issues, data privacy, potential over-reliance on AI.

PRISMA checklist

Section and Topic	Item #	Checklist item	Location where item is reported
**TITLE**			
Title	1	Identify the report as a systematic review.	Page 1
**ABSTRACT**			
Abstract	2	See the PRISMA 2020 for Abstracts checklist.	Page 2
**INTRODUCTION**			
Rationale	3	Describe the rationale for the review in the context of existing knowledge.	Pages 4 & 5
Objectives	4	Provide an explicit statement of the objective(s) or question(s) the review addresses.	Page 6
**METHODS**			
Eligibility criteria	5	Specify the inclusion and exclusion criteria for the review and how studies were grouped for the syntheses.	Pages 29 & 30
Information sources	6	Specify all databases, registers, websites, organisations, reference lists and other sources searched or consulted to identify studies. Specify the date when each source was last searched or consulted.	Page 29
Search strategy	7	Present the full search strategies for all databases, registers and websites, including any filters and limits used.	Page 29
Selection process	8	Specify the methods used to decide whether a study met the inclusion criteria of the review, including how many reviewers screened each record and each report retrieved, whether they worked independently, and if applicable, details of automation tools used in the process.	Page 30
Data collection process	9	Specify the methods used to collect data from reports, including how many reviewers collected data from each report, whether they worked independently, any processes for obtaining or confirming data from study investigators, and if applicable, details of automation tools used in the process.	Pages 30 & 31
Data items	10a	List and define all outcomes for which data were sought. Specify whether all results that were compatible with each outcome domain in each study were sought (e.g. for all measures, time points, analyses), and if not, the methods used to decide which results to collect.	Page 31
	10b	List and define all other variables for which data were sought (e.g. participant and intervention characteristics, funding sources). Describe any assumptions made about any missing or unclear information.	Page 31
Study risk of bias assessment	11	Specify the methods used to assess risk of bias in the included studies, including details of the tool(s) used, how many reviewers assessed each study and whether they worked independently, and if applicable, details of automation tools used in the process.	Pages 31
Effect measures	12	Specify for each outcome the effect measure(s) (e.g. risk ratio, mean difference) used in the synthesis or presentation of results.	N/A
Synthesis methods	13a	Describe the processes used to decide which studies were eligible for each synthesis (e.g. tabulating the study intervention characteristics and comparing against the planned groups for each synthesis (item #5)).	N/A
	13b	Describe any methods required to prepare the data for presentation or synthesis, such as handling of missing summary statistics, or data conversions.	N/A
	13c	Describe any methods used to tabulate or visually display results of individual studies and syntheses.	N/A
	13d	Describe any methods used to synthesize results and provide a rationale for the choice(s). If meta-analysis was performed, describe the	N/A

Section and Topic	Item #	Checklist item	Location where item is reported
		model(s), method(s) to identify the presence and extent of statistical heterogeneity, and software package(s) used.	
	13e	Describe any methods used to explore possible causes of heterogeneity among study results (e.g. subgroup analysis, meta-regression).	N/A
	13f	Describe any sensitivity analyses conducted to assess robustness of the synthesized results.	N/A
Reporting bias assessment	14	Describe any methods used to assess risk of bias due to missing results in a synthesis (arising from reporting biases).	Pages 31
Certainty assessment	15	Describe any methods used to assess certainty (or confidence) in the body of evidence for an outcome.	Pages 31
**RESULTS**			
Study selection	16a	Describe the results of the search and selection process, from the number of records identified in the search to the number of studies included in the review, ideally using a flow diagram.	Page 6
	16b	Cite studies that might appear to meet the inclusion criteria, but which were excluded, and explain why they were excluded.	N/A
Study characteristics	17	Cite each included study and present its characteristics.	Page 9
Risk of bias in studies	18	Present assessments of risk of bias for each included study.	Page 10
Results of individual studies	19	For all outcomes, present, for each study: (a) summary statistics for each group (where appropriate) and (b) an effect estimate and its precision (e.g. confidence/credible interval), ideally using structured tables or plots.	N/A
Results of syntheses	20a	For each synthesis, briefly summarise the characteristics and risk of bias among contributing studies.	N/A
	20b	Present results of all statistical syntheses conducted. If meta-analysis was done, present for each the summary estimate and its precision (e.g. confidence/credible interval) and measures of statistical heterogeneity. If comparing groups, describe the direction of the effect.	N/A
	20c	Present results of all investigations of possible causes of heterogeneity among study results.	N/A
	20d	Present results of all sensitivity analyses conducted to assess the robustness of the synthesized results.	N/A
Reporting biases	21	Present assessments of risk of bias due to missing results (arising from reporting biases) for each synthesis assessed.	Pages 10 & 11
Certainty of evidence	22	Present assessments of certainty (or confidence) in the body of evidence for each outcome assessed.	Pages 12 & 13
**DISCUSSION**			
Discussion	23a	Provide a general interpretation of the results in the context of other evidence.	Page 20
	23b	Discuss any limitations of the evidence included in the review.	Page 22, 23,
	23c	Discuss any limitations of the review processes used.	Page 20
	23d	Discuss implications of the results for practice, policy, and future research.	Page 26, 27

Section and Topic	Item #	Checklist item	Location where item is reported
**OTHER INFORMATION**			
Registration and protocol	24a	Provide registration information for the review, including register name and registration number, or state that the review was not registered.	Page 29
	24b	Indicate where the review protocol can be accessed, or state that a protocol was not prepared.	Page 29
	24c	Describe and explain any amendments to information provided at registration or in the protocol.	N/A
Support	25	Describe sources of financial or non-financial support for the review, and the role of the funders or sponsors in the review.	Page 1 & 33
Competing interests	26	Declare any competing interests of review authors.	Page 1 & 33
Availability of data, code and other materials	27	Report which of the following are publicly available and where they can be found: template data collection forms; data extracted from included studies; data used for all analyses; analytic code; any other materials used in the review.	Page 33
